# Persistent Organic Pollutants in Norwegian Men from 1979 to 2007: Intraindividual Changes, Age–Period–Cohort Effects, and Model Predictions

**DOI:** 10.1289/ehp.1206317

**Published:** 2013-09-05

**Authors:** Therese Haugdahl Nøst, Knut Breivik, Ole-Martin Fuskevåg, Evert Nieboer, Jon Øyvind Odland, Torkjel Manning Sandanger

**Affiliations:** 1 Department of Community Medicine, University of Tromsø, Tromsø, Norway; 2 NILU-Norwegian Institute for Air Research, Fram Centre, Tromsø, Norway; 3 University Hospital of North Norway, Tromsø, Norway; 4 NILU-Norwegian Institute for Air Research, Kjeller, Norway; 5 Department of Chemistry, University of Oslo, Oslo, Norway; 6 Department of Biochemistry and Biomedical Sciences, McMaster University, Hamilton, Ontario, Canada

## Abstract

Background: Longitudinal monitoring studies of persistent organic pollutants (POPs) in human populations are important to better understand changes with time and age, and for future predictions.

Objectives: We sought to describe serum POP time trends on an individual level, investigate age–period–cohort effects, and compare predicted polychlorinated biphenyl (PCB) concentrations to measured values.

Methods: Serum was sampled in 1979, 1986, 1994, 2001, and 2007 from a cohort of 53 men in Northern Norway and analyzed for 41 POPs. Time period, age, and birth cohort effects were assessed by graphical analyses and mixed-effect models. We derived the predicted concentrations of four PCBs for each sampling year using the CoZMoMAN model.

Results: The median decreases in summed serum POP concentrations (lipid-adjusted) in 1986, 1994, 2001, and 2007 relative to 1979 were –22%, –52%, –54%, and –68%, respectively. We observed substantial declines in all POP groups with the exception of chlordanes. Time period (reflected by sampling year) was the strongest descriptor of changes in PCB-153 concentrations. Predicted PCB-153 concentrations were consistent with measured concentrations in the study population.

Conclusions: Our results suggest substantial intraindividual declines in serum concentrations of legacy POPs from 1979 to 2007 in men from Northern Norway. These changes are consistent with reduced environmental exposure during these 30 years and highlight the relation between historic emissions and POP concentrations measured in humans. Observed data and interpretations are supported by estimates from the CoZMoMAN emission-based model. A longitudinal decrease in concentrations with age was evident for all birth cohorts. Overall, our findings support the relevance of age–period–cohort effects to human biomonitoring of environmental contaminants.

Citation: Nøst TH, Breivik K, Fuskevåg OM, Nieboer E, Odland JØ, Sandanger TM. 2013. Persistent organic pollutants in Norwegian men from 1979 to 2007: intraindividual changes, age–period–cohort effects, and model predictions. Environ Health Perspect 121:1292–1298; http://dx.doi.org/10.1289/ehp.1206317

## Introduction

The use of persistent organic pollutants (POPs) in agriculture and industry increased markedly beginning in the 1930s [Arctic Monitoring and Assessment Programme (AMAP) 2004]. As concerns for the detrimental effects of POPs on the environment and human health increased, measures to reduce or eliminate the production and use of POPs were initiated in many countries from the 1970s onward (AMAP 1998). Consequently, global emissions of legacy POPs have largely followed the same trends. Reduced use and emissions of legacy POPs were followed by declining POP concentrations in air and biota (e.g., Hung et al. 2010; Rigét et al. 2010), which has led to reduced human exposure. Accordingly, declining concentrations of most banned compounds have been reported in the few available human longitudinal POP studies (Hagmar et al. 2006; Hovinga et al. 1992; Høyer et al. 2000; Tee et al. 2003; Vo et al. 2008).

POP concentrations have frequently been reported to be positively associated with age (Hardell et al. 2010; Rylander et al. 1997; Wolff et al. 2005) and to birth cohorts (Bjerregaard et al. 2001; Perry et al. 2005; Wolff et al. 2007) in human cross-sectional studies. In such studies, age and birth cohort effects are confounded (Glenn 2003; Quinn and Wania 2012). In longitudinal epidemiologic studies, age effects reflect differences in risk factors between age groups, period effects reflect temporal changes in factors that affect all individuals in a population, and birth cohort effects reflect generation-specific influences (Glenn 2003; Holford 1991; Palmore 1978). The interdependence of age, period, and cohort effects produces mutual confounding in time-trend studies. This has previously not been considered in empirical studies of POP time trends.

A person’s lifetime environmental exposure to any POP (i.e., the intensity and duration of individual environmental exposure) depends on birth year relative to the time of peak environmental concentrations. Increasing age is associated with physiological changes (e.g., changes in body composition and metabolism) and changes in dietary patterns that may influence both the intake and the elimination of POPs. Environmental exposures and dietary habits also differ among birth cohorts and contribute to differences in the duration and intensity of exposures to specific POPs. Finally, periodic patterns reflect changes in environmental POP concentrations related to historic emissions and environmental persistence, in addition to temporal trends in dietary intakes.

The present study was based on five repeated measurements of serum POPs concentrations during 1979–2007 in a cohort of 53 men from Northern Norway. Our primary aims were to describe intraindividual changes in POP concentrations and composition and to investigate age–period–cohort (APC) effects. In addition, we compared measured POPs concentrations and observed APC patterns to predictions based on the time-variant CoZMoMAN model (Breivik et al. 2010). Use of emission-based mechanistic modeling in combination with the unique empirical data available for the study population provides insight into the relationship between environmental emissions and observed concentrations in humans.

## Subjects and Methods

*Study population and subject selection*. Five repeated population surveys in the Tromsø study (summarized by Jacobsen et al. 2012) took place in the municipality of Tromsø in Northern Norway, in 1979, 1986–1987 (hereafter referred to as 1986), 1994–1995 (1994), 2001, and 2007–2008 (2007). Of 60 randomly selected men, 53 had sufficient sample volumes in ≥ 3 sampling years (11 missing samples were randomly distributed across sampling years). In total, the present analyses comprised 254 serum samples from 53 men. Birth year and body mass index information was extracted from questionnaires. The study was approved by the Regional Committees for Medical Research Ethics. Participation was voluntary and all participants provided informed consent. Serum samples were stored at –70°C until analysis.

*Analytical methodology*. All contaminant analyses were performed during 2011 at the laboratories of the Norwegian Institute for Air Research (NILU) and the University Hospital of Northern Norway (UNN). Serum samples were extracted and analyzed for polychlorinated biphenyls (PCBs) and organochlorine pesticides [chlordanes, hexachlorohexanes (HCHs), hexachlorobenzene (HCB), 1,1,1-trichloro-2,2-bis(*p*-chlorophenyl)ethane (*p,p*´-DDT) and its metabolites (DDTs), and toxaphenes]. A complete list of the individual POPs is provided in Supplemental Material, Table S1.

Extraction and cleanup. Methods for extraction and cleanup were modified from Sandanger et al. (2003, 2007). We weighed serum samples [mean 0.92 g (range, 0.24–1.12 g)] and added 26 ^13^C-labeled internal standards, deionized water saturated with ammonium sulfate (1 mL), methanol (2 mL), and hexane (6 mL) to the samples. Each serum mixture was vortexed and shaken for 1 hr. The samples were centrifuged (1,200 rpm, 6 min) in an Eppendorf 5702R centrifuge (Eppendorf, Hamburg, Germany), and the supernatant hexane was pipetted off. The extraction protocol was repeated with 6 mL of hexane, and the hexane supernatants were combined and evaporated to 0.5 mL in a heated vacuum evaporation unit. We performed subsequent cleanup using solid-phase extraction columns (Florisil, 1 g, deactivated) in an automated liquid handling system. Each column was prewashed with hexane/dichloromethane (DCM) (12 mL; 9:1 wt/wt) and hexane (12 mL) before the extract was applied. Analytes were subsequently eluted with hexane/DCM (12 mL; 9:1 wt/wt), evaporated to 0.2 mL, transferred to a GC-vial, further reduced to ~ 30 μL by gentle nitrogen flow, and recovery standard (octachloronaphtalene) was then added.

Instrumental analysis. Chlorinated pesticides (excluding DDTs) (1-μL injection volume) were analyzed on an Agilent 7890A gas chromatograph (GC) (Agilent Technologies Europe, Boeblingen, Germany) equipped with a 5975c mass spectrometer (MS) (instrumental details were described previously by Hansen et al. 2010). We operated the MS in selected ion monitoring (SIM) and negative chemical ionization (NCI) modes at 160°C. All POPs were analyzed with the GC temperature program used by Hansen et al. (2010) with the exception of the toxaphenes, which were analyzed separately under the following conditions: 70°C (3 min), 25°C min^–1^ to 180°C (0 min), and 15°C min^–1^ to 280°C (5 min).

We analyzed PCBs and DDTs on the same GC as described above but with a Quattro Micro triple quadrupole MS (Waters Corporation, Manchester, UK). Injector settings, GC column, carrier gas, and the temperature program were as described above for pesticide analyses. The MS operated in MS/MS (MRM) mode with an electron ionization source at 220°C. Argon (~ 0.23 Pa) was the collision gas. Information regarding ion transitions has been published previously (Pitarch et al. 2007).

Lipid determination. Analyses of triglycerides, phospholipids, free cholesterol, and total cholesterol were determined enzymatically by Unilab Analyse AS, Tromsø, Norway, and a summed lipid concentration was calculated according to the equation proposed by Akins et al. (1989).

*Quality assurance and sample integrity*. Quality control in POP and lipid analyses. To assess laboratory-derived sample contamination and method accuracy and reproducibility, we processed blanks (*n* = 9) and standard reference materials (SRMs) [SRM® 1958 (*n* = 9) and 1957 (*n* = 9), both from the National Institute of Standards and Technology, Gaithersburg, MD, USA] along with the samples. Results for SRMs indicated analytical uncertainties within ± 20% of assigned values (within ± 5% for many compounds). The NILU laboratory routinely participates in the international AMAP Ring Test for Persistent Organic Pollutants in Human Serum and has performed well (within ± 20% of assigned values). Concomitantly, summed lipid concentrations in the test samples (*n* = 10) were within a 15% deviation from assigned values. [Ring test results are available from the Institut national de santé publique du Québec (2013).]

Mean recoveries of internal standards were 81%, 83%, 77%, 79%, and 56% for the 1979, 1986, 1994, 2001, and 2007 samples, respectively. The internal standard recovery for the 2007 samples was low in one sample preparation batch (53% of the 2007 samples); however, there was no association between recoveries and concentrations (data not shown). Recoveries were < 30% in three samples but were not excluded from statistical analyses because they did not constitute extreme observations nor did they deviate in model diagnostic plots. We rejected results when their isotopic mass ratios deviated by > 20% from the quantification standards. PCB-138/163, PCB-47/49, and PCB-28/31 coeluted, and we summed their concentrations. The limits of detection (LODs) were software-generated and corresponded to signal-to-noise ratios of 3. Because consistent amounts of β–HCH and *oxy*-chlordane were measured in blanks, we subtracted mean blank concentrations for these compounds from all samples.

Estimation of desiccation and lipid degradation. To correct for spuriously high POP concentrations caused by evaporation during long-term storage, serum sodium (Na^+^) was measured and used to adjust lipid and POP concentrations. In samples with Na^+^ concentrations > 165 mmol/L (3% of samples), lipid and POP concentrations were adjusted by the ratio [Na^+^]_mean_/[Na^+^]_sample_ (Krieger et al. 1994). Na^+^ determinations were conducted at UNN using an ion-selective electrode method.

Total cholesterol and triglycerides were measured previously and, after adjusting current results for desiccation, the past and current measurements deviated < 10% and correlations between the two measurements increased (data not shown).

*Time-variant model simulations of PCBs in serum*. Simulations of lipid-normalized serum concentrations of PCBs 118, 138, 153, and 180 for 1979, 1986, 1994, 2001, and 2007 were carried out using the time-variant multimedia mechanistic CoZMoMAN model (Breivik et al. 2010). Previous CoZMoMAN model predictions for PCB concentrations and their temporal changes in women were within the ranges of measured concentrations (Breivik et al. 2010; Quinn and Wania 2012). Simulations were performed assuming time-variant emission scenarios (Quinn et al. 2011). In general, model parameters were set as outlined by Breivik et al. (2010). Specifically, we assumed that trends and concentrations in the environment and food chains in Northern Norway are similar to the trends for Sweden and parts of Southeastern Norway that were used for model development, except for the dietary input parameters. We assumed that fish consumption in the population of older men from Northern Norway was higher than the original model input; therefore, we derived separate predictions based on average Norwegian fish consumption and three categories of higher fish consumption (for detailed information, see Supplemental Material, pp. 3–4 and Table S2). Model predictions for PCB-153 for the birth years 1930, 1935, 1940, and 1945 (within the range of birth years of the study subjects) were obtained, assuming that either all of the birth cohorts had equal fish consumption or that fish consumption differed among birth cohorts, with the earliest birth cohort consuming the most fish, and the latest cohort the least.

*Data treatment and statistical methods*. We performed statistical analyses using R, version 2.13.1 (R Foundation for Statistical Computing, Vienna, Austria). Statistical significance was defined as *p* < 0.05. All POP results were lipid-adjusted and log_e_-transformed in the statistical analyses. We excluded three samples from 1979 with high (> median + 2SD) and three samples from 1994 with low lipid-adjusted concentrations (< median – 2SD) from statistical models in order to obtain the most appropriate model estimates; however, analyses including these samples and when performed on wet weight concentrations gave the same main results (data not shown). Lipid concentrations were missing for one sample, and thus the numbers of observations in the statistical analyses were 51, 51, 45, 48, and 52 for the five time points.

We calculated summed POP concentrations based on lipid-adjusted concentrations of compounds with > 60% detection; for values below LOD, we used the individual concentration estimates. Summary statistics for compounds with detection frequencies between 20% and 80% were calculated for each sampling year using the Kaplan–Meier method with the NADA package for R according to Helsel (2005).

Spearman’s ρ values were calculated for correlations. We used the Wilcoxon signed rank test to test differences in POP concentrations between sampling years and the Kruskal–Wallis rank sum test to test differences between birth year groups (categorized according to quartiles) in each sampling year.

We assessed APC effects for serum concentrations of PCB-153 using age and birth cohort groups categorized according to quartiles. We used mixed-effect models (lme4 package for R) that included a random slope for sampling year and subject-specific random terms (to allow subject-specific random variation) to estimate periodic changes in PCB-153 concentrations and potential age-specific and birth cohort–specific effects. Despite collinearity, models used to assess APC effects must include all three time parameters (Palmore 1978); therefore, we used mixed-effects models with two parameters modeled as fixed effects and the third modeled as a random effect (Ding et al. 2007). We also assessed body mass index as a fixed effect. Confidence intervals (CIs) for coefficients were obtained post hoc (glht in multcomp R package). We used Akaike’s information criterion (AIC) to compare models, and the nonparametric Friedman’s test to test differences across all measurements.

A graphical examination of APC effects for PCB-153 was carried out by plotting all six combinations of the three time factors according to Ahacic et al. (2012) to assess longitudinal patterns (i.e., concentrations according to birth cohort and sampling period or age), time-lag patterns (concentrations according to age and sampling period or birth cohort), or cross-sectional patterns (concentrations according to sampling period and age or birth cohort).

## Results

*Characteristics of study participants*. Median ages at the first and last sampling were 43 and 71 years, respectively (Table 1). The median birth year was 1936 (range, 1925–1950). The number of subjects in each age and birth cohort quartile is listed in Supplemental Material, Table S3.

**Table 1:** Descriptive age statistics of study participants measured in 1979, 1986, 1994, 2001, and 2007 (all male).

Age	1979 (*n* = 51)	1986 (*n* = 51)	1994 (*n* = 45)	2001 (*n* = 48)	2007 (*n* = 52)
Median	43	50	58	65	71
Minimum	29	36	44	51	57
Maximum	54	61	69	76	82

**Figure 1:**
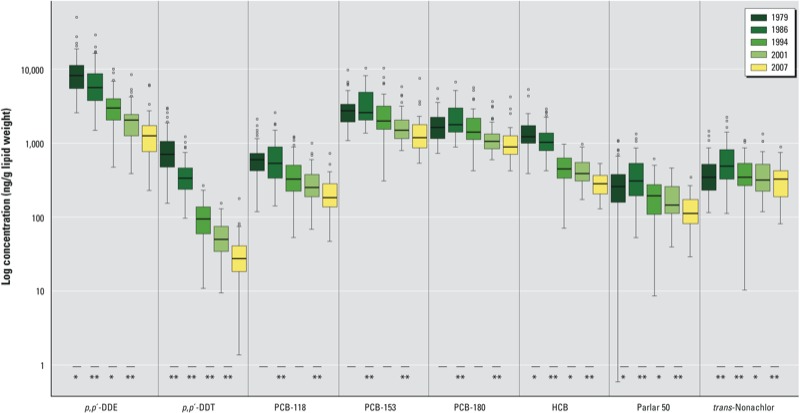
Concentrations (ng/g lipid, log_e_ scale) of selected POPs analyzed in repeated serum samples of men (*n* = 51, 51, 45, 48, and 52 in 1979, 1986, 1994, 2001, and 2007, respectively) from Northern Norway. *p,p´*-DDE, 1,1-dichloro-2,2-bis(*p*-chlorophenyl)ethylene. Parlar 50 represents toxaphenes, and *trans*-nonachlor the chlordanes. Boxes represent the 25th–75th percentiles, horizontal lines represent the median, whiskers indicate 1.5 times the length of the interquartile range above and below the 75th and 25th percentiles, respectively, and outliers are represented as data points. *p < 0.05, and **p < 0.001 for comparisons between pairs of consecutive sampling years.

**Figure 2:**
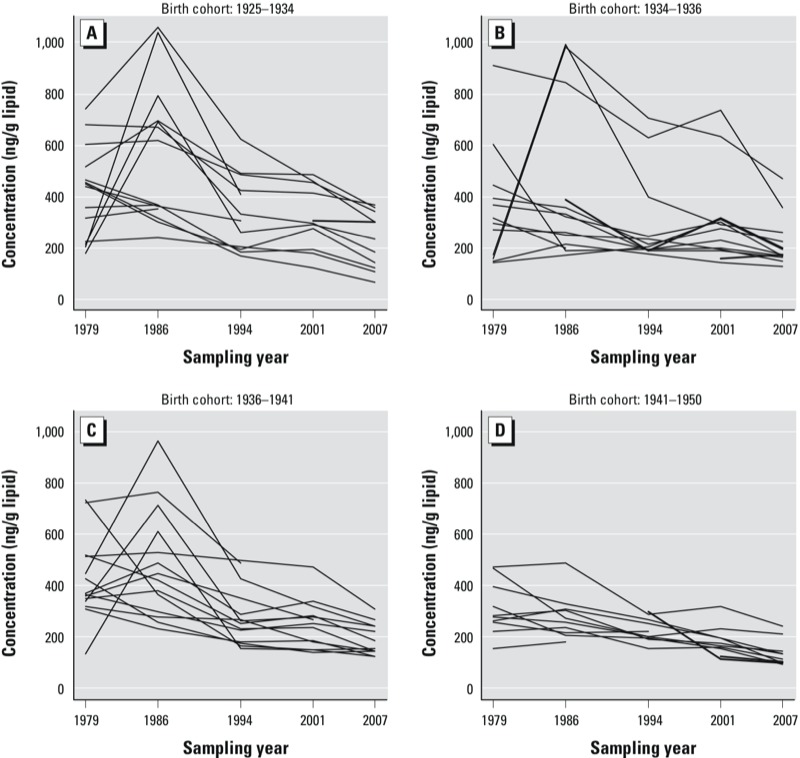
Individual trend lines for PCB-153 serum concentrations (ng/g lipid) measured in 1979, 1986, 1994, 2001, and 2007 in 53 men from Northern Norway, according to birth year quartile. (*A*) 1925–1934, (*B*) 1934–1936, (*C*) 1936–1941, and (*D*) 1941–1950.

*Intraindividual changes in POP concentrations*. Serum POP concentrations in each sampling year are presented for selected compounds in Figure 1 (see Supplemental Material, Table S4 for complete data for all analyzed POPs). The median individual decreases in summed POP concentrations in 1986, 1994, 2001, and 2007 relative to the median concentration in 1979 were –22%, –52%, –54%, and –68%, respectively. Substantial declines were observed for all POPs with the exception of chlordanes [e.g., *trans-*nonachlor (Figure 1)]. Overall, decreases were observed from 1979 in concentrations of HCHs, HCB, *c*-chlordane, DDTs, and most penta-chlorinated PCBs (PCBs 99, 101, 105, 118, and 123) and hexa-chlorinated PCBs (PCB 128, 141, 149, 153, and 167). Declining trends were exponential for many POPs, especially for *p,p´-*DDT (*R*^2^ = 0.78 for fitted exponential trend line, data not shown). Concentrations of chlordanes (except *c*-chlordane), mirex, toxaphenes, and hepta- and octa-chlorinated PCBs (PCBs 170, 180, 187, and 194) initially increased from 1979 to 1986, and then declined in subsequent years. Concentration differences across all sampling years were significant for all POPs (Friedman’s test, *p* < 0.001); however, the absolute differences in the concentrations of chlordanes were small (e.g., 47 and 45 ng/g lipid adjusted for *trans-*nonachlor in 1979 and 2007, respectively). Within individuals, the concentrations of most POPs were higher in 1979 than in 2007, but time trends varied among individuals, as shown for PCB-153 (Figure 2; individuals grouped according to birth year).

**Figure 3:**
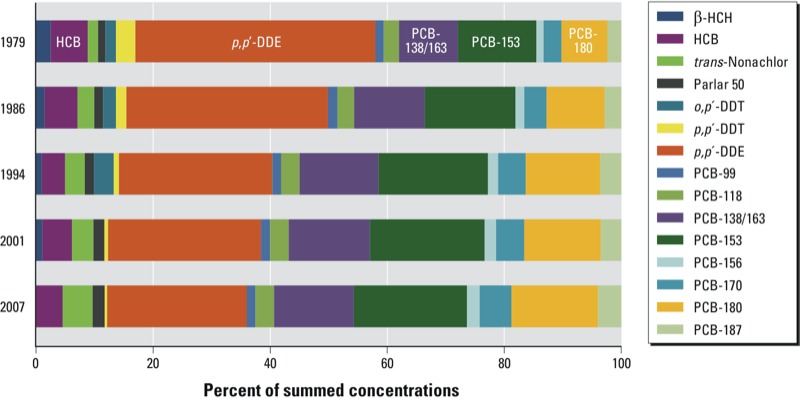
Relative contributions of individual POPs that accounted for > 1% of summed POPs in 1979 in serum from men (*n* = 51, 51, 45, 48, and 52 in 1979, 1986, 1994, 2001, and 2007, respectively) in Northern Norway.

Relative contributions of individual POPs to summed POP concentrations (as a percentage) are shown in Figure 3. Clearly, 1,1-dichloro-2,2-bis(*p*-chlorophenyl)ethylene (*p,p´-*DDE); PCBs 153, 138/163, and 180; and HCB were the most prominent (67–73% of sum in the different sampling years). *p,p´*-DDE/*p,p´*-DDT ratios were 12, 20, 29, 41, and 55 for 1979, 1986, 1994, 2001, and 2007, respectively.

*POP correlations across sampling years*. The correlations of concentrations (lipid-adjusted) in any pair of consecutive sampling years increased across the study period for most compounds. Concentrations correlated significantly between 1986 and 1994, 1994 and 2001, and 2001 and 2007 for PCB-153 (ρ = 0.72, 0.80, and 0.87, respectively, all *p* < 0.001), and between all sampling years for *p,p´*-DDE (ρ = 0.44, 0.79, 0.87, and 0.89 between 1979 and 1986, 1986, and 1994, 1994 and 2001, and 2001 and 2007, respectively, all *p* < 0.002).

*Intercompound correlations*. In 1979, correlations were strong (ρ > 0.85) between PCB-153 and *oxy*-chlordane, mirex, and PCBs 99, 118, 138/163, 156, 157, 167, 170, 180, 183, 187, 189, and 194. The correlations between PCB-153 and other POPs were also strong in the subsequent sampling years (ρ > 0.85; *n* = 13, 8, 10, and 9 compounds for 1986, 1994, 2001, and 2007, respectively), yet slightly weaker over time.

*Predicted PCB concentrations*. Measured and predicted concentrations (nanograms per gram lipid) of PCBs 118, 138, 153, and 180 from CoZMoMAN simulations for a 1935 birth cohort of men are presented in Supplemental Material, Figure S1 for the 5 sampling years according to four different assumptions regarding fish consumption. Model predictions were generally consistent with measured concentrations, especially for PCBs 153 and 180, but overestimated concentrations of PCB-118 and underestimated concentrations of PCB-138. Predicted trends (assuming equal fish consumption among birth cohorts) for PCB-153 concentrations for men born in 1930, 1940, and 1945 (see Supplemental Material, Figure S2) showed similar trends among birth years.

*Estimated APC effects on changes in PCB-153 concentrations*. Estimates from mixed-effect models of PCB-153 concentrations in the different sampling years, with age, period, and birth cohort modeled as fixed predictors or as random effects (accounting for subject–specific variation) are shown in Table 2. The best fitting model included period and birth cohort as predictors, and age as a random effect. Estimates for the period effects (i.e., changes according to sampling year) were generally consistent among models. Including body mass index for all sampling years did not improve the model (data not shown). Additional models that specified interactions or nonlinear effects (as product or cubic terms, respectively) were not possible (they did not converge).

**Table 2:** Mixed-effect model estimatesa [coefficients (95% CIs)] of changes in PCB-153 concentrations (ng/g lipid) during 1979–2007 among 53 men from Northern Norway, with age, calendar period, and birth cohort as predictors.

Predictor	Model 1: period only (fixed effects)	Model 2: period and age (fixed effects)	Model 3: period and age (fixed effects) plus birth cohort (random effect)^*b*^	Model 4: period and birth cohort (fixed effects)	Model 5: period and birth cohort (fixed effects) plus age (random effect)^*b*^
Period^*c*^
1979	Referent	Referent	Referent	Referent	Referent
1986	51 (–68, 221)	72 (–69, 284)	82 (–71, 311)	67 (–98, 326)	22 (–104, 202)
1994	–85 (–156, 12)	–66 (–159, 73)	–82 (–175, 54)	–82 (–179, 62)	–117 (–191, –16)
2001	–95 (–163, –2)	–76 (–172, 74)	–93 (–189, 52)	–84 (–181, 57)	–119 (–194, –17)
2007	–160 (–210, –90)	–150 (–224, –30)	–175 (–245, –65)	–163 (–238, –52)	–190 (–251, –103)
Age (years)^*d*^
29–47	—	Referent	Referent	—	—
47–57	—	–32 (–122, 94)	–31 (–122, 94)	—	—
57–66	—	–35 (–138, 117)	–27 (–133, 126)	—	—
66–82	—	–24 (–144, 167)	–4 (–129, 184)	—	—
Birth cohort^*e*^
1925–1934	—	—	—	Referent	Referent
1934–1936	—	—	—	–73 (–210, 170)	–61 (–199, 173)
1936–1941	—	—	—	–24 (–180, 249)	–17 (–171, 244)
1941–1950	—	—	—	–139 (–246, 50)	–149 (–251, 26)
AIC^*f*^	154	157	176	151	137
All models included a subject-specific random term and a random slope for sampling year; age and birth cohort variables were divided into quartiles. ^***a***^Coefficients are backtransformed from log-estimates of fixed effect variables and are in units of ng/g lipid. ^***b***^Variables were added to models as random terms to allow for random variation in individuals. ^***c***^Coefficients express change for PCB-153 concentrations (ng/g lipid) across sampling years, with 1979 as the reference period category. ^***d***^Coefficients express change in PCB-153 concentrations (ng/g lipid) across age quartiles, with the youngest age group (29–47 years) as the reference category. ^***e***^Coefficients express change in PCB-153 concentrations (ng/g lipid) across birth cohort quartiles with the oldest birth cohort group (1925–1934) as the reference category. ^***f***^Lower Akaike’s information criterion numbers indicate better model fit when comparing models.					

**Figure 4:**
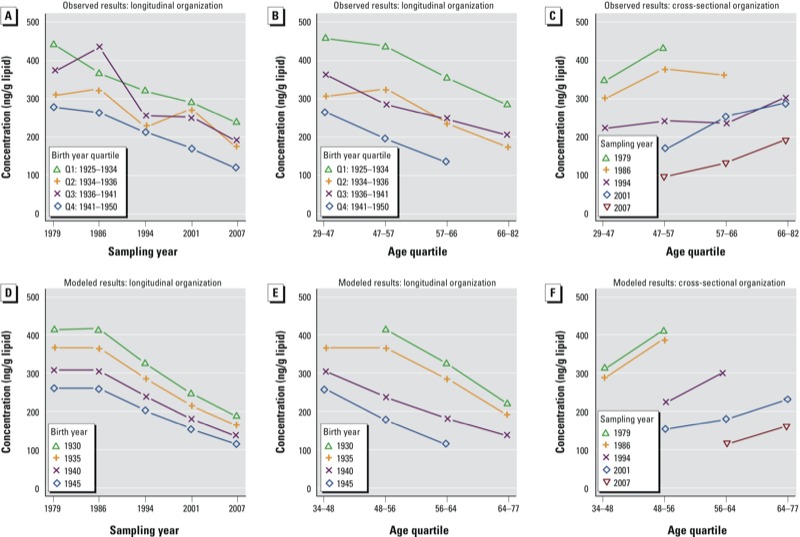
APC plots showing observed (*A–C*) and predicted serum PCB-153 concentrations (*D–F*, using the CoZMoMAN model assuming higher fish consumption in earlier birth cohorts). *A* and *D* show longitudinal variation among birth cohorts according to sampling period, *B* and *E* show longitudinal variation among birth cohorts according to age quartile (*Q*), and *C* and *F* cross-sectional variation among sampling periods according to age quartile. Data points indicate ng/g lipid-adjusted concentrations (median concentrations are displayed in *A–C*). Differences between birth cohorts in *A* were significant in 1986 and 2007 (Kruskal–Wallis rank sum test, *p* < 0.05).

Figure 4 shows selected plots of a graphical examination of APC effects. The period effect is apparent as decreasing PCB-153 concentrations across the sampling years and as subjects aged (Figure 4A,B). Although differences in PCB-153 concentrations among birth cohort quartiles across sampling years were not significant for some sampling years (Figure 4A), the most recently born cohort (1941–1950) appeared to have the lowest concentrations, and the earliest cohort (1925–1934) had the highest. Within sampling years, concentrations increased with age and decreased in more recent birth cohorts (Figure 4C; see also Supplemental Material, Figure S3C). Finally, within age groups, concentrations decreased according to sampling period and birth year (see Supplemental Material, Figure S3A and B, respectively). Overall, the graphical examination suggests clear period effects and additional birth cohort effects, whereas age effects appear relatively weak. APC patterns based on predicted PCB-153 concentrations (Figure 4D–F; see also Supplemental Material, Figure S3D–F) were generally consistent with patterns based on measured values.

## Discussion

*Intraindividual changes in POP concentrations from 1979 to 2007*. Overall, our findings suggest that POP concentrations decreased during 1979–2007 in men from Northern Norway. Average summed POP concentrations in 2007 were one third of concentrations measured in 1979. The majority of POP concentrations declined from 1979, although median concentrations of some compounds peaked in 1986 (e.g., PCBs 170, 180, and 194). Peak PCB-153 concentrations were measured in 1979 and 1986, confirming this period as the years of highest human exposure, a feature which was also reproduced by CoZMoMAN (see Supplemental Material, Figure S1). We also observed large individual variability during these years, as could be expected.

The downward trends in serum concentrations likely reflect declining environmental concentrations due to reduced emissions during the same time period. This is in accordance with previous findings for environmental and human POP concentrations in Europe (AMAP 1998; Bignert et al. 1998). Our findings indicate that serum concentrations of DDTs peaked before PCBs, which is consistent with emission estimates for DDTs (Li and Macdonald 2005) and PCBs (Breivik et al. 2010). The delay in global emissions of PCBs could be due to the long lifetime of PCB-containing products (e.g., transformers, capacitors) (Breivik et al. 2007). The declines of many POPs, especially *p,p*´-DDT, were exponential and indicated nonlinear rates of decrease across the study period. The concentrations of some compounds were unchanged or decreased only slightly (chlordanes and mirex), emphasizing the need to include these compounds in monitoring studies. Correlations of POPs in any pair of consecutive sampling years became stronger during the study period, possibly due to reduced concentrations and reduced variability of exposures over time.

Our results suggest that regulatory measures to reduce the manufacture and use of POPs during the 1970s and 1980s had rapid impacts not only on environmental concentrations (AMAP 1998), but also on human exposures. Substantial reductions in human intake rates relative to elimination rates are suggested.

*PCB-153 concentrations and aAPC effects*. Time period had the strongest influence on PCB-153 concentrations based on both the graphical examination and the mixed-effect analyses. Although the range of birth years (1925–1950) was relatively narrow, the mixed-effect analyses also suggested an additional influence of birth cohort. This may reflect differences in cumulative exposure and dietary patterns among birth cohorts, consistent with associations between PCB-153 and age or birth cohort that have been reported based on cross-sectional studies (Bjerregaard et al. 2001; Hardell et al. 2010; Perry et al. 2005; Rylander et al. 1997; Wolff et al. 2005, 2007).

Changes in dietary intakes (Quinn et al. 2012) and body mass (Wolff et al. 2007) must also be considered when evaluating time trends in human POP concentrations. When model simulations included the assumption of higher fish consumption in earlier birth cohorts (in accordance with the available dietary information and intergenerational dietary differences in Quinn et al. 2012), we observed consistent patterns of predicted and observed PCB-153 concentrations according to birth cohort (Figure 4A,D). Although predictions were not based on individual dietary information, CoZMoMAN model estimates were consistent with observed birth cohort patterns. This supports the use of mechanistic modeling in hypothesis testing and illustrates that an understanding of temporal trends in emissions and of confounded time factors is relevant for POP monitoring studies.

*Changes in relative concentrations of POPs*. The relative concentrations of POPs in human serum changed over time, both between and within POP groups. The majority of POPs evaluated were highly correlated with PCB-153 concentrations during all sampling years, supporting the use of PCB-153 as a marker compound for many legacy POPs, although it is important to note that correlations with PCB-153 concentrations weakened over time.

Proportions relative to the sum of all POPs decreased for *p,p*´-DDE (from 37% in 1979 to 21% in 2007) and increased for the sum of PCB 138, 153, and 180 (from 30% in 1979 to 44% in 2007). A steeper decline in concentrations for *p,p*´-DDE compared with PCB-153 has been reported previously based on human longitudinal studies (Hagmar et al. 2006; Hovinga et al. 1992; Vo et al. 2008). Because the *p,p*´-DDE/*p,p*´-DDT ratio is sensitive to recent exposure to *p,p*´-DDT (Anda et al. 2007), its increase from 12 in 1979 to 55 in 2007 suggests that exposure to *p,p*´-DDT was markedly reduced. The relative contribution of PCBs (and especially the higher–chlorinated congeners) to summed POPs increased, and thus PCBs might be expected to dominate future organochlorine POP burdens. Overall, the relative changes in POP concentrations may be explained by differences in emission histories, environmental persistence, exposure sources, and elimination rates.

*Comparisons to other longitudinal studies*. Clearly, age distribution and time of sampling relative to historic POP emissions must be considered when comparing longitudinal POP trends. The concentrations and temporal changes in PCB-153, *p,p*´-DDE, and HCB in our study population were similar to findings for younger Swedish men sampled in 1991 and 2001 (Hagmar et al. 2006). Measured concentrations were lower and peaked later in our study population than POPs concentrations measured in two longitudinal cohorts in the Great Lakes area [Hovinga et al. 1992 (sampled in 1982 and 1989); Tee et al. 2003 (sampled in 1980, 1990, and 1994)]. These similarities and differences may reflect geographical differences in environmental exposures during the same time period, in addition to different dietary patterns.

*Evaluation of time-variant model predictions*. We challenged the CoZMoMAN mechanistic model with observations in order to evaluate its performance. Overall, the model’s predictions were in reasonable agreement with temporal changes in median measured concentrations from 1979 to 2007, especially for PCB-153. The observed overestimation of PCB-118 by the CoZMoMAN model has also been reported by Czub and McLachlan (2004), who suggested that it could be due to an incorrect assumption of zero metabolism of this congener in humans. The coelution of PCB-138 and PCB-163 in chromatograms is likely the reason for the apparent underestimation of PCB-138. The model did predict an initial increase of PCB-180 during the early 1980s consistent with our empirical results, although the observed decline afterwards was slightly steeper than predicted.

*Study limitations*. Although statistical approaches to APC effects have been much discussed in the literature, no consensus has been reached (Glenn 2003). Currently, suitable *p*-values in mixed-effect models cannot be calculated in the statistical software. Potential interactions between the time factors could not be considered in the mixed-effect model analysis, and our ability to examine APC effects was limited by the small numbers of men in some age groups.

Individual dietary information was not available for the model parameterization; therefore, we used estimates of average and high fish consumption in Norwegian populations to reflect the potential range of fish consumption in the study population instead.

## Conclusions

Longitudinal declines in legacy POPs in serum samples from Norwegian men during 1979–2007 are consistent with reduced environmental exposures in this period. Our adaptation of methods to assess APC effects based on biomonitoring data is novel and suggests that calendar time (i.e., period effects) had a major influence on observed concentrations, although birth cohort differences were also indicated. Predicted concentrations and time trends for PCB-153 were consistent with those measured. The use of APC analysis and emission-based modeling in human biomonitoring enhances our understanding of the relationship between temporal trends in human POP burdens and historical emissions.

## Supplemental Material

(791 KB) PDF
