# Supplementary material for: Persistent Organic Pollutants in Norwegian Men from 1979 to 2007: Intraindividual Changes, Age–Period–Cohort Effects, and Model Predictions
Source: Environ Health Perspect. 2013 Sep 5;121(11-12):1292–8. doi: 10.1289/ehp.1206317 (PMC3855502; doi:10.1289/ehp.1206317)
Supplement: (791 KB) PDF [file ehp.1206317.s001.508.pdf]

Environmental Health Perspectives doi:10.1289/ehp.1206317

SUPPLEMENTAL MATERIAL

Persistent Organic Pollutants in Norwegian Men from 1979 to 2007: Intraindividual Changes, Age–Period–Cohort Effects, and Model Predictions

Therese Haugdahl Nøst,[1,2,3] Knut Breivik,[4,5] Ole-Martin Fuskevåg,[3] Evert Nieboer,[1,6] Jon Øyvind Odland,[1] and Torkjel Manning Sandanger[1,2]

[1]Department of Community Medicine, University of Tromsø, Tromsø, Norway; [2]NILU-Norwegian Institute for Air Research, Fram Centre, Tromsø, Norway; [3]University Hospital of North Norway, Tromsø, Norway; [4]NILU-Norwegian Institute for Air Research, Kjeller, Norway; [5]Department of Chemistry, University of Oslo, Oslo, Norway; [6]Department of Biochemistry and Biomedical Sciences, McMaster University, Hamilton, Ontario, Canada.

Table of contents

Supplemental Material, Table S1 Page 2

Information on dietary parameters used in CoZMoMAN modeling Page 3

Supplemental Material, Table S2 Page 4

Supplemental Material, Table S3 Page 5

Supplemental Material, Table S4 Page 6

Supplemental Material, Figure S1 Page 8

Supplemental Material, Figure S2 Page 9

Supplemental Material, Figure S3 Page 10

Supplemental Material, References Page 11

Supplemental Material, Table S1: List of compounds analyzed in blood samples.

Analyzed compounds	Abbreviation or IUPAC No.
Polychlorinated biphenyls (PCBs)	Congeners 18, 28, 33, 47/49, 52, 99, 101, 105, 118, 123, 128, 138/163, 141, 149, 153, 156, 157, 167, 170, 180, 183, 187, 189, 194
Hexachlorocyclohexanes (HCHs)	α-HCH, β-HCH, γ-HCH
Hexachlorobenzene	HCB
Chlordanes (CHLs)	*trans*- and *cis*-Chlordane, *oxy*-Chlordane, *trans*- and *cis*-Nonachlor
Mirex	
DDT and metabolites (DDTs)	1,1,1-trichloro-2,2-bis(*p*-chlorophenyl)ethane (*p,p′*-DDT)
	1,1,1-trichloro-2-(*o*-chlorophenyl)-2-(*p*-chlorophenyl)ethane (*o,p′*-DDT)
	1,1-dichloro-2,2-bis(*p*-chlorophenyl)ethene (*p,p′*-DDE)
	1,1-dichloro-2-(*o*-chlorophenyl)-2-(*p*-chlorophenyl)ethene (*o,p′*-DDE)
	1,1-dichloro-2,2-bis(*p*-chlorophenyl)ethane (*p,p′*-DDD)
	1,1-dichloro-2-(*o*-chlorophenyl)-2-(*p*-chlorophenyl)ethane (*o,p′*-DDD)
Toxaphenes	Parlar 26 (B8–1413) and 50 (B9–1679)

Supplemental Material, Information on dietary input parameters used in CoZMoMAN.

The original description of dietary habits is based on the food consumption of the Swedish population (Czub and McLachlan 2004). Specifically, scenarios for the ingestion of fish, beef and dairy products were developed as a function of time from 1930 onwards. However, dietary habits of the current study population (i.e., men in Northern Norway) are likely to differ from the Swedish population with respect to fish consumption in particular. While historical dietary information from study subjects were insufficient, information on fish consumption in the Norwegian population is only available for the years after 1995 (Norwegian Directorate of Health 2010). While the general Swedish population eats fish 1.7 times/week, the general Norwegian population does so 2.3 times/week (Bergsten 2004). Most detailed information on fish consumption in the Norwegian population was available for 2000, and the dietary input to CoZMoMAN reflected this information by adjusting the original time-variant dietary parameterization to the numeric information for year 2000 (see Table S2: average fish consumption scenario for Norway (FC_{avr})).

An even higher rate of fish consumption was expected for the study population, compared to the general Norwegian population. High age, male sex and living in Northern Norway are all factors associated with high fish consumption (Alexander et al. 2006; Bergsten 2004; Johansson and Solvoll 1999). Simulations were therefore carried out based on four different scenarios for fish intake, representing the average Norwegian fish intake (as detailed above), as well as for three additional scenarios representing increased consumption of fish (FC1-FC3, see Table S2).

The highest fish consumption category (FC3) data were obtained by adjusting the fish consumption to a 95% percentile of total daily fish intake in 2000 (Bergsten 2004) (see Table S2), with a corresponding reduction in the intake of meat. Two additional categories between the average Norwegian fish intake and the highest were calculated (see Table S2). Furthermore, the proportion of fish intake comprised of lean fish is high in the Norwegian population (2/3 of all fish consumption) (Alexander et al. 2006) and the herring/cod ratio used as a fat fish/lean fish indication in the model was consequently changed from 0.75/0.25 in the Swedish dietary parameters to 0.35/0.65 for the study population. Meat consumption was adjusted corresponding to changes in fish consumption in all categories. Consumption of dairy products in the study population as a function of time was assumed to be similar to that of the Swedish population.

In model simulations presented in Figure 4 and Supplemental Material, Figure S3, the fish consumption categories for the birth cohorts 1930, 1935, 1940 and 1945 were assumed to be F3, F2, F1 and F_{avr}, respectively.

Supplemental Material, Table S2: Fish consumption categories used in CoZMoMAN.

Fish consumption category	Abbreviation	Original CoZMoMAN parameterization (g ww/day in 2000)	In this article (g ww/day in 2000)
Average	FC_{avr}	92	124
Higher 1	FC1		156
Higher 2	FC2		202
Higher 3	FC3		248

Supplemental Material, Table S3: Number of subjects in each quartile of the variables age and birth year.

	Age quartiles				Birth year quartiles			
**Sampling year**	29-47	47-57	57-66	66-82	1925-1934	1934-1936	1936-1941	1941-1950
1979	42	9	0	0	14	12	14	11
1986	17	29	5	0	15	12	14	10
1994	5	18	19	3	12	10	13	10
2001	0	7	29	12	12	13	13	10
2007	0	1	13	38	15	13	13	11

Supplemental Material, Table S4: Concentrations (ng/g lipid) of 41 compounds[a] analyzed in serum samples of men (N = 51, 51, 45, 48 and 52 in 1979, 1986, 1994, 2001 and 2007, respectively) in the Tromsø study.

	1979 N=51				1986 N=51				1994 N=45				2001 N=48				2007 N=52			
**Compound**	Median (Range)	AM	% > LOD[b]	LOD[c]	Median (Range)	AM	% > LOD[b]	LOD[c]	Median (Range)	AM	% > LOD[b]	LOD[c]	Median (Range)	AM	% > LOD[b]	LOD[c]	Median (Range)	AM	% > LOD[b]	LOD[c]
α-HCH	3.7 (0.6-23)	4.3	100	0.4	1.8 (0.8-13)	2.2	100	0.5	- (0.1-0.8)	0.4	38	0.4	0.4 (0.1-1.2)	0.6	48	0.6	-	-	8	0.6
β-HCH[d]	67 (13-190)	72	100	8.4	34 (3.9-100)	40	96	8.5	13 (1.1-50)	14	79	7.8	13 (0-48)	14	67	9.5	- (1.4-25)	8.8	30	9.4
γ-HCH	2.2 (0.7-16)	3	79	1.7	- (0.7-4.8)	1.7	38	1.7	-	-	10	1.6	-	-	4	1.9	-	-	0	1.9
HCB	170 (34-420)	180	100	3.2	130 (71-410)	150	100	3.2	53 (31-130)	61	100	2.9	62 (20-130)	64	100	3.6	40 (15-85)	43	100	3.6
t-chlordane	0.4 (0.2-1.9)	0.5	53	0.5	0.6 (0.2-2.3)	0.7	63	0.5	0.3 (0.2-1)	0.5	46	0.5	0.3 (0.2-1.2)	0.5	38	0.6	-	-	17	0.6
c-chlordane	-	-	15	2.3	-	-	10	2.3	-	-	2	2.1	-	-	0	2.6	-	-	0	2.6
oxy-chlordane[e]	25 (6.5-56)	25	98	2	27 (11-100)	35	100	2	18 (9.1-49)	21	98	1.8	21 (5.9-63)	24	100	2.2	18 (4.2-53)	20	100	2.2
t-nonachlor	47 (5.4-150)	51	100	0.8	67 (23-230)	80	100	0.8	45 (19-130)	53	100	0.7	45 (14-170)	60	100	0.9	45 (9.5-140)	50	100	0.9
c-nonachlor	12 (1.1-36)	12	100	1.5	14 (5.4-55)	19	100	1.5	11 (4.5-33)	13	98	1.4	13 (3.7-40)	15	100	1.7	11 (3-35)	13	100	1.7
Mirex	6.6 (1.5-17)	7.5	98	1.7	8.9 (3.7-33)	11	100	1.6	7.3 (2.5-22)	9.2	100	1.6	8.7 (3.5-25)	9.8	100	1.6	7.5 (2.8-21)	8.6	100	1.6
Parlar No. 26	18 (2.7-77)	21	96	0.6	18 (5.4-87)	27	100	0.6	9.4 (3.2-33)	12	98	0.5	9.2 (1.5-31)	12	100	0.7	7.6 (1.9-21)	8.6	100	0.7
Parlar No. 50	34 (5.3-130)	39	96	1.7	35 (8.2-160)	52	98	1.7	21 (9.5-61)	27	98	1.6	23 (4.6-65)	28	100	2	18 (3.9-50)	20	98	1.8
p,p′-DDD	16 (2.5-73)	21	91	6	9.4 (2.9-67)	13	79	5.6	- (1.1-14)	4.7	23	5.2	-	-	6	6.3	-	-	6	6.3
o,p′-DDT	48 (1.1-87)	4	85	4	51 (1.1-45)	11	79	2.4	45 (0.3-6.5)	34	23	2.3	-	-	13	2.8	-	-	8	2.4
p,p′-DDT	89 (21-440)	110	98	4.7	41 (17-160)	51	100	3.2	12 (4.5-33)	13	98	3	7.7 (1.5-27)	8.5	81	3.7	3.8 (0.2-25)	5.3	60	3.3
p,p′-DDE	1100 (230-3400)	1200	100	4.1	800 (250-4500)	920	100	4.2	350 (100-1400)	390	100	3.7	320 (60-980)	320	100	4.8	210 (29-770)	210	100	4.6
o,p′-DDE	1 (0.3-5.6)	1.6	53	1.6	- (0.1-5.5)	1	38	1.2	-	-	8	1.1	-	-	0	1.4	-	-	2	1.2
PCB 18	5.4 (1.2-84)	8	85	2.6	-	-	10	2.7	-	-	0	2.4	- (0.5-6.1)	2.8	31	3	2.6 (0.5-9.1)	3.1	51	3
PCB 28	5.5 (1.3-43)	6.7	92	2.7	2.9 (1.1-72)	5.2	56	2.9	- (0.7-4.9)	2.3	15	2.6	- (1.2-11)	2.6	21	3.2	- (1.3-6.1)	2.7	26	3.1
PCB 33	0.8 (0.2-30)	2	58	1	-	-	17	0.8	-	-	2	0.8	-	-	19	0.9	- (0.1-2.3)	0.7	28	1
PCB 47/49	1.4 (0.5-8.4)	1.6	89	1.1	1 (0.3-73)	2.4	71	1.3	0.4 (0.2-1.4)	0.5	40	0.7	0.9 (0.2-4)	1.2	73	0.7	0.7 (0.2-2.8)	0.8	74	0.9
PCB 52	3.8 (0.9-30)	5.8	58	4.2	- (1.3-73)	5.9	42	4.6	-	-	10	3.8	-	-	15	4.7	-	-	19	4.5
PCB 99	38 (12-110)	45	100	0.9	38 (16-130)	46	100	0.9	20 (10-52)	25	100	0.7	19 (7-44)	22	100	1.1	13 (4-34)	15	100	0.9
PCB 101	6.1 (1.1-19)	7.5	94	3.4	5.1 (2-74)	8	81	3.3	2.1 (1.5-10)	3.3	50	3.1	2.3 (1.8-7)	3.1	46	3.5	- (1.2-7.7)	2.7	25	3.7
PCB 105	16 (4.4-55)	20	98	1.8	15 (5-79)	21	100	1.4	9.1 (2.5-27)	11	98	1	8.2 (1.4-25)	9.5	98	1.4	6 (1.3-17)	6.7	100	1.3
PCB 118	70 (19-230)	83	100	3.4	66 (20-330)	88	100	2.9	42 (14-120)	49	100	2.9	39 (7.9-130)	44	100	3.2	28 (6.1-82)	32	100	3.2
PCB 123	1.7 (0.1-7.1)	1.9	66	1.8	1.5 (0.1-3.6)	2.1	69	1.4	1 (0.1-3.6)	1.3	63	1	- (0-3.6)	1.2	44	1.4	- (0-2.9)	0.8	28	1.3
PCB 128	2.8 (0.2-11)	3.1	77	1.7	2.5 (0.3-14)	3.3	81	1.5	1.5 (0.3-5.6)	1.8	65	1.2	- (0.2-4.1)	1.3	33	1.6	-	-	11	1.4

	1979 N=51				1986 N=51				1994 N=45				2001 N=48				2007 N=52			
**Compound**	Median (Range)	AM	% > LOD[b]	LOD[c]	Median (Range)	AM	% > LOD[b]	LOD[c]	Median (Range)	AM	% > LOD[b]	LOD[c]	Median (Range)	AM	% > LOD[b]	LOD[c]	Median (Range)	AM	% > LOD[b]	LOD[c]
PCB 138/163	270 (110-670)	310	100	3.5	280 (130-770)	350	100	3.3	180 (120-490)	210	100	3.1	170 (77-450)	190	100	3.8	120 (45-310)	140	100	4
PCB 141	-	-	40	2.1	-	-	23	1.8	-	-	15	1.6	-	-	10	1.9	-	-	2	2.5
PCB 149	2.6 (0.5-8.7)	3.2	85	1.9	2.2 (0.3-11)	3	71	1.7	0.7 (0.1-4.8)	1.3	44	1.4	- (0.1-3.1)	1.4	42	1.8	-	-	11	2.3
PCB 153	360 (130-910)	380	100	4.6	360 (180-1100)	450	100	4.6	250 (160-700)	290	100	4.5	240 (120-740)	260	100	5.3	170 (67-470)	200	100	5.2
PCB 156	33 (12-78)	35	100	1.2	35 (17-92)	42	100	1	23 (14-61)	27	100	0.9	24 (10-69)	26	100	1.1	19 (8-52)	21	100	1
PCB 157	6.1 (1.9-14)	6.6	98	1.1	6.9 (2.8-19)	8.2	100	0.9	4.9 (1.4-12)	5.5	96	0.8	4.9 (1.7-15)	5.6	100	1	3.8 (1.3-9.5)	4.3	100	0.9
PCB 167	11 (3.3-32)	13	100	1.1	11 (4-44)	14	100	1	7.4 (2.5-23)	8.9	100	0.8	7.1 (1.3-23)	7.9	100	1	5.7 (1-17)	6.1	100	0.9
PCB 170	82 (32-180)	87	100	1.5	89 (47-250)	110	100	1.4	63 (38-160)	72	100	1.2	59 (31-160)	66	100	1.6	48 (26-130)	53	100	1.4
PCB 180	210 (82-540)	230	100	4.1	230 (130-740)	290	100	3.9	170 (110-460)	200	100	3.9	160 (87-470)	190	100	4.6	130 (66-370)	150	100	4.6
PCB 183	21 (9.5-49)	23	100	1.1	19 (7.5-66)	25	100	1	13 (7-37)	15	100	0.9	10 (3.9-32)	12	100	1.1	7.5 (1.9-23)	8.5	100	1.1
PCB 187	64 (22-140)	67	100	1.3	66 (31-230)	81	100	1.2	48 (29-120)	54	100	1	43 (24-110)	49	100	1.3	35 (15-88)	39	100	1.2
PCB 189	3.7 (1.2-8.1)	4	98	1.2	4 (1.9-13)	5	100	1.1	3.2 (1.4-8.2)	3.6	94	0.9	3.3 (1.3-9)	3.5	100	1.2	2.5 (0.8-7)	2.7	92	1.2
PCB 194	24 (9.1-79)	27	100	1.4	28 (15-130)	34	100	1.3	22 (14-78)	26	100	1.1	20 (11-55)	24	100	1.5	18 (8.7-50)	20	100	1.3

[a]For compound abbreviations, see Table S1. Censored summary statistics are presented for compounds with detection frequencies less than 90%: *α*-, *β*-, *γ*-HCH, *t*-chlordane, PCB congeners 18, 28, 33, 47/49, 52, 101, 123, 128, 149, *p,p'*-DDD, *o,p'*-DDT and *o,p'*-DDE.

[b]% > LOD = Percentage of sample in which analyte was detected.

[c]LOD = Limit of detection (average of sample-specific LODs).

[d]Presented concentrations reflect the subtraction of average blank values of 107 pg before calculating wet-weight concentrations.

[e]Presented concentrations reflect the subtraction of average blank values of 43 pg before calculating wet-weight concentration.

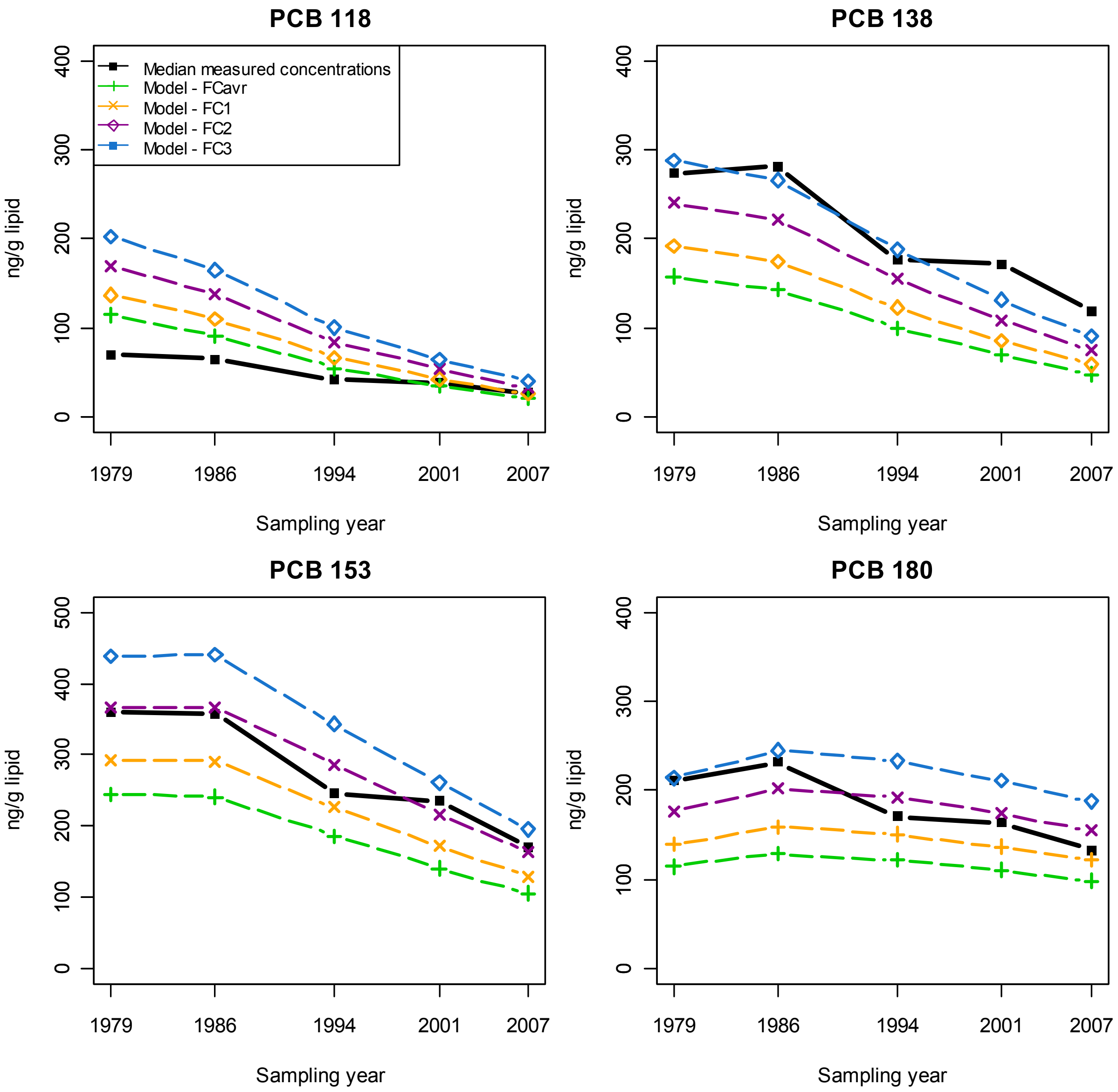

Supplemental Material, Figure S1: Model predictions of concentrations (ng/g lipid) of PCBs 118, 138, 153, and 180 in a male 1935 birth cohort shown along with median measured concentrations in 1979, 1986, 1994, 2001 and 2007. Separate predictions for average Norwegian fish consumption (FC_{avr}) and three categories of higher fish intake rates are presented (FC1-FC3).

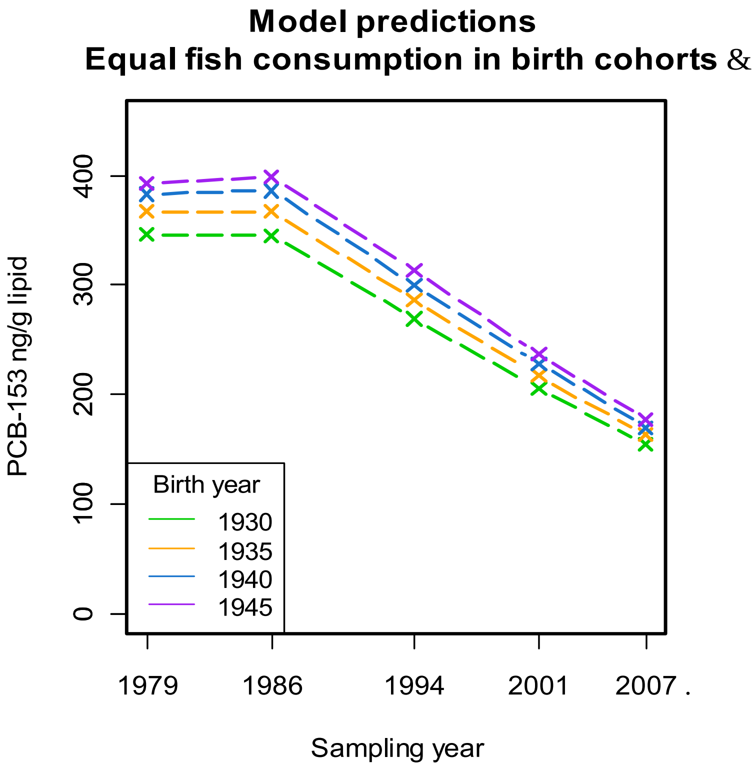

Supplemental Material, Figure S2: Model predictions of concentrations (ng/g lipid) of PCB-153 are displayed for the 1930, 1935, 1940 and 1945 male birth cohorts. All cohorts are assumed to have equal fish consumption (FC2).

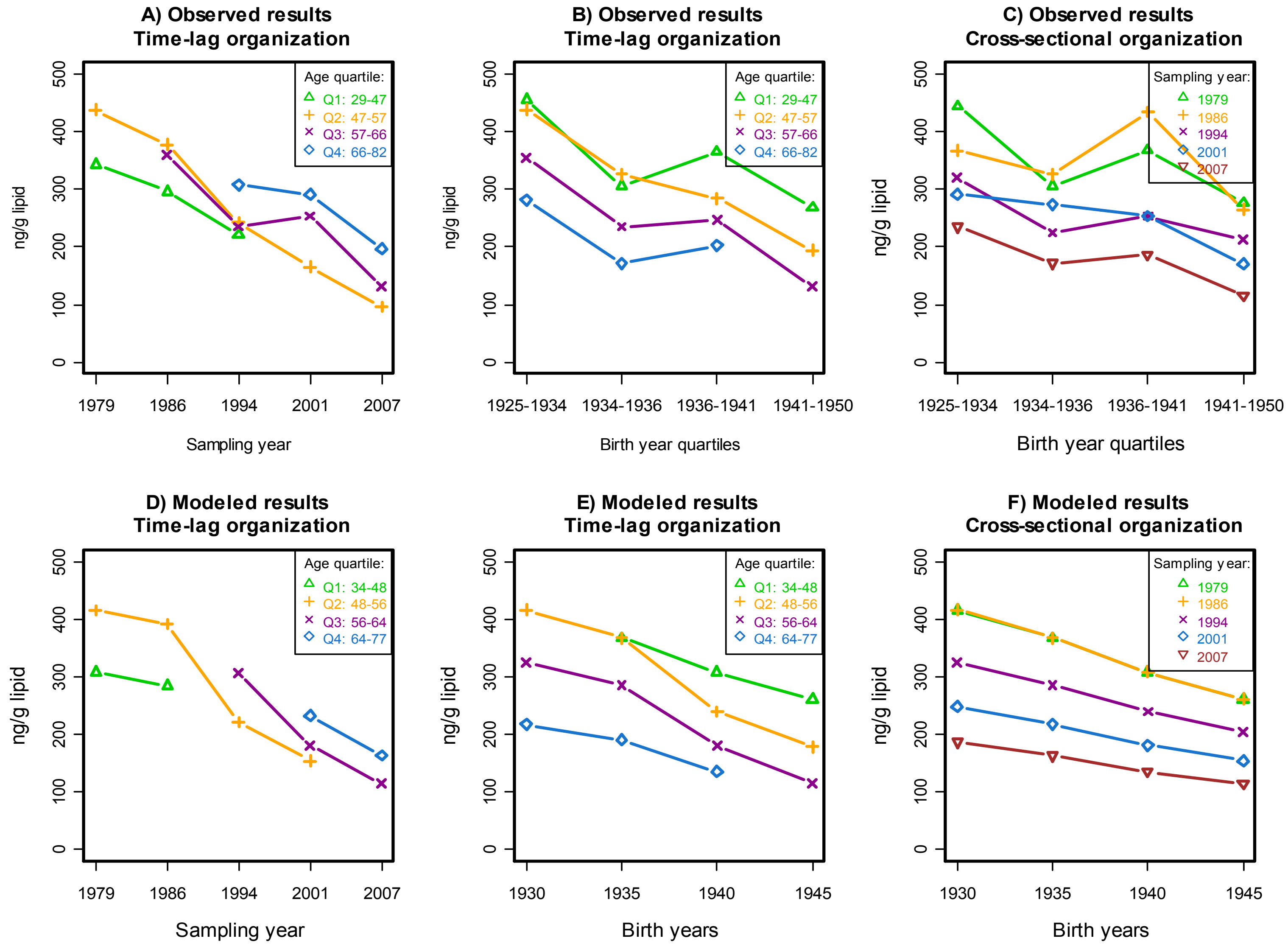

Supplemental Material, Figure S3: Remaining plots of graphical APC examination in PCB-153 concentrations. This figure complements Figure 4 in the main text. A and D depict time-lag variation among age groups according to sampling period, B and E time-lag variation among age groups according to birth cohort group, and C and F cross-sectional variation among sampling periods according to birth cohort group.

Supplemental Material, References

Alexander J, Frøyland L, Hemre G, Koster Jacobsen B, Lund E, Meltzer H. 2006. Et helhetssyn på fisk og annen sjømat i norsk kosthold. [in Norwegian] Oslo, Norway. Norwegian Scientific Committee for Food Safety. Available from: www.vkm.no/dav/a2805d6a8c.pdf

Bergsten C. 2004. Fish and game study, part B. (The consumption of foods that may be important when assessing the dietary intake of mercury, cadmium and PCB/dioxins, with a focus on population groups living on the coast and in the inland of Norway). [in Norwegian] Oslo, Norway. Norwegian Food Safety Authority. Available from: www.mattilsynet.no/mattilsynet/multimedia/archive/00016/Fisk_og_vilt__Fish_a_16664a.pdf

Czub G, McLachlan MS. 2004. A food chain model to predict the levels of lipophilic organic contaminants in humans. Environ Toxicol Chem 23(10): 2356-2366.

Johansson L, Solvoll KN. 1999. Norkost 1997. Landsomfattende kostholdsundersøkelse blant menn og kvinner i alderen 16-79 år. [in Norwegian] Report No.: 2/1999. Oslo, Norway. National Council on Nutrition and Physical Activity. Available from: www.helsedirektoratet.no/folkehelse/ernering/tall-og-undersokelser/Documents/norkost-1997.pdf

Norwegian Directorate of Health. 2010. Utviklingen i norsk kosthold. Matforsyningsstatistikk og Forbruksundersøkelser. [in Norwegian] Report No.: IS-1873. Oslo, Norway.Available from: http://www.helsedirektoratet.no/publikasjoner/utviklingen-i-norsk-kosthold-2010-stor-utgave/Publikasjoner/utviklingen-i-norsk-kosthold2010–stor-utgave.pdf
